# FZD7 regulates BMSCs-mediated protection of CML cells

**DOI:** 10.18632/oncotarget.6742

**Published:** 2015-12-23

**Authors:** Na Liu, Shaolei Zang, Yan Liu, Yingqiao Wang, Wei Li, Qiang Liu, Min Ji, Daoxin Ma, Chunyan Ji

**Affiliations:** ^1^ Department of Hematology Qilu Hospital, Shandong University, Ji'nan 250012, China; ^2^ Medical Research Center, Shandong Provincial Qianfoshan Hospital, Shandong University, Ji'nan 250014, China

**Keywords:** FZD7, CML, BMSCs, imatinib sensitivity, Wnt signaling pathway

## Abstract

Inspite of effective treatment with imatinib (IM), chronic myeloid leukemia (CML) is still an incurable disease. Some patients became refractory because of IM resistance. Bone marrow mesenchymal stem cells (BMSCs) have been implicated a role in promoting CML cells' resistance against IM treatment. The detailed molecular mechanisms, however, remain largely unknown. In this study, we found that BMSCs increased the expression of FZD7 and activated Wnt/β-catenin signaling pathway in CML cells. BMSCs from CML patients showed increased efficiency to accelerate CML cell proliferation, enhance the drug resistance of K562 cells and up-regulate the expression of FZD7. Antagonism of FZD7 expression by shRNA significantly suppressed proliferation and increased IM sensitivity of CML cells co-cultured with BMSCs cells. Our findings suggest that FZD7, involved in canonical Wnt signaling pathway, plays a critical role in mediating BMSCs-dependent protection of CML cells, and potentially provide a novel therapeutic target for CML.

## INTRODUCTION

Chronic myeloid leukemia (CML) is a clonal myeloproliferative disease which originates from a primitive hematopoietic stem cells (HSCs) transformed by BCR-ABL oncogene. The deregulated tyrosine kinase activity of BCR-ABL protein leads to increased proliferation and reduced apoptosis of undifferentiated myeloid cells [1]. Imatinib mesylate (IM) and other tyrosine kinase inhibitors (TKIs) have been highly efficient in treatment of CML. However, a significant proportion of patients do not obtain expected effectiveness, while some other CML patients become refractory to further treatment [2]. Moreover, cessation of drug treatment leads to disease recurrence in most CML patient. Minimal residual disease (MRD), retained in patients even when they reach complete remission, may be the source of relapse in CML patients after TKI discontinuation [3, 4].

Some evidences suggest that disease relapse and treatment resistance in CML patients are largely due to the protection of leukemia cells by various components of the bone marrow microenvironment. Especially indirect communication through extracellularly secreted growth factors [5] and direct contact between leukemia cells and bone marrow mesenchymal stem cells (BMSCs) appear to be essential for CML cells survival and chemoresistance [6]. However, the signaling pathway that mediates the interaction of CML cells with BMSCs and causes the chemotherapy resistance of CML cells is largely unexplored.

Wnt/b-catenin signaling is highly evolutionarily conserved and is involved in embryogenesis and the maintenance of homeostasis in tissues by regulating cellular processes such as proliferation, differentiation, survival, and angiogenesis [7]. The Wnt/b-catenin signaling is initiated by binding of Wnt ligands to frizzled transmembrane receptors (FZD) and low-density lipoprotein receptor-related proteins (LRPs), resulting in stabilization of β-catenin, which subsequently translocates to the nucleus. Imported β-catenin forms transcriptional complex with TCF/LEF to activate transcription of downstream target genes, such as *CD44*, *cyclin D1* and *c-Myc, Survivin*, and *Trib2* [8–11]. Studies have showed that BMSCs enhance nuclear translocation and transcriptional activity of b-catenin in CML cells [12]. However, the molecular basis that how Wnt signaling activity in CML cells is regulated by BMSCs remains obscure.

In this study, we found that BMSCs could increase the expression of Frizzled-7 (FZD7) and subsequently activate Wnt/b-catenin signaling pathway in CML cells. Co-cultured CML cells with BMSCs showed up-regulated FZD7 expression, increased cell proliferation and decreased drug sensitivity, which could be reversed by FZD7 knockdown with shRNA. Our findings suggest that FZD7 plays a critical role in mediating BMSCs-promoted CML cells proliferation and drug resistance through Wnt/b-catenin signaling pathway. Therefore, our work provide a foundation of FZD7 to be a novel therapy target for CML.

## RESULTS

### FZD7 along with β-catenin and its downstream melocules was up-regulated in CML cells following contact with BMSCs

**Figure 1:**
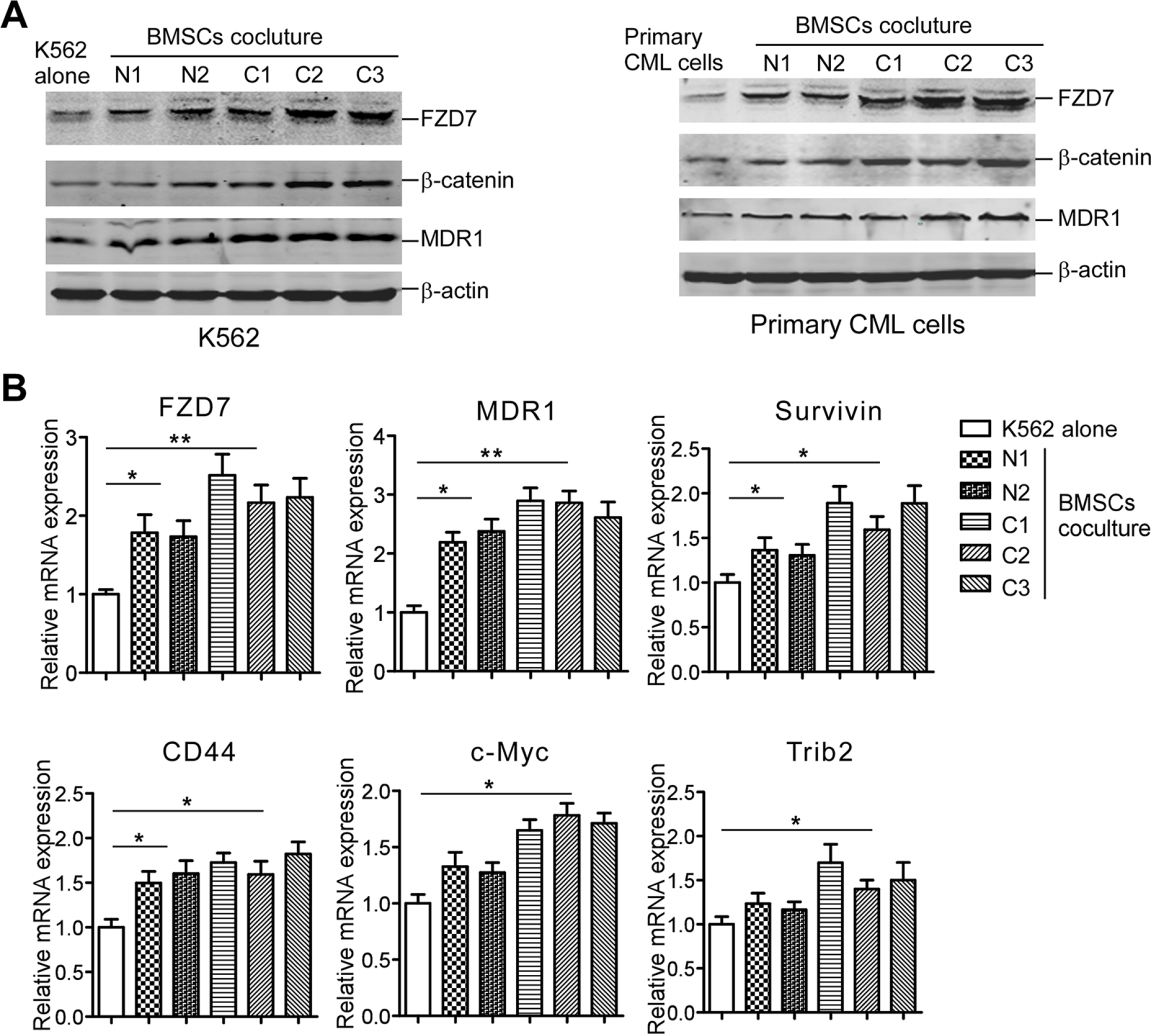
BMSCs induce FZD7 expression along with β-catenin, and Wnt downstream moleculars in co-cultured CML cells K562 cells or primary CML cells were cultured solely or co-cultured with BMSCs (derived from 3 initially diagnosed CML patients and 2 healthy donors) for 2 days. (**A**) Western blot was used to detect the expression of FZD7, β-catenin, and downstream Wnt downstream molecular-MDR1. “N” stands for “Normal” and “C” stands for “CML”. (**B**) Real-time RT-PCR analysis was used to detect the expression of FZD7 and downstream Wnt downstream molecular-MDR1, Survivin, CD44, c-Myc, and Trib2. Values represent means ± S.E. (*n* = 3). *P < 0.05. **P < 0.01.

Studies showed that co-culturing with BMSCs significantly inhibited CML cells' apoptosis and protected CML cells from TKIs exposure [12]. To explore the key molecules that mediate the interaction between BMSCs and CML cells, especially those facilitate BMSCs-dependent CML preservation, we built a system where CML cells were co-cultured with BMSCs derived from 3 initially diagnosed CML patients or 2 healthy donors. Western blot analysis showed that co-culturing with normal BMSCs or CML-BMSCs sharply increased FZD7, β-catenin, and Wnt downstream target MDR1 expression in K562 cells (Figure 1A. left) and primary CML cells (Figure 1A, right), respectively. Interestingly, the BMSCs from CML patiens exhibited higher efficiency to promote the expression levels of these proteins. In agreement with the western blot data, real-time RT-PCR showed that co-culture with normal MSCs and CML-MSCs sharply increased Wnt signaling target genes *FZD7*, *MDR1*, *Survivin*, *CD44*, *c-Myc*, and *Trib2* mRNA expression in K562 cells (Figure 1B). These results indicated that FZD7 might take part in the crosstalk between CML cells and BMSCs.

### Up-regulation of FZD receptors was observed in CD34^+^ cells of CML patients

**Figure 2:**
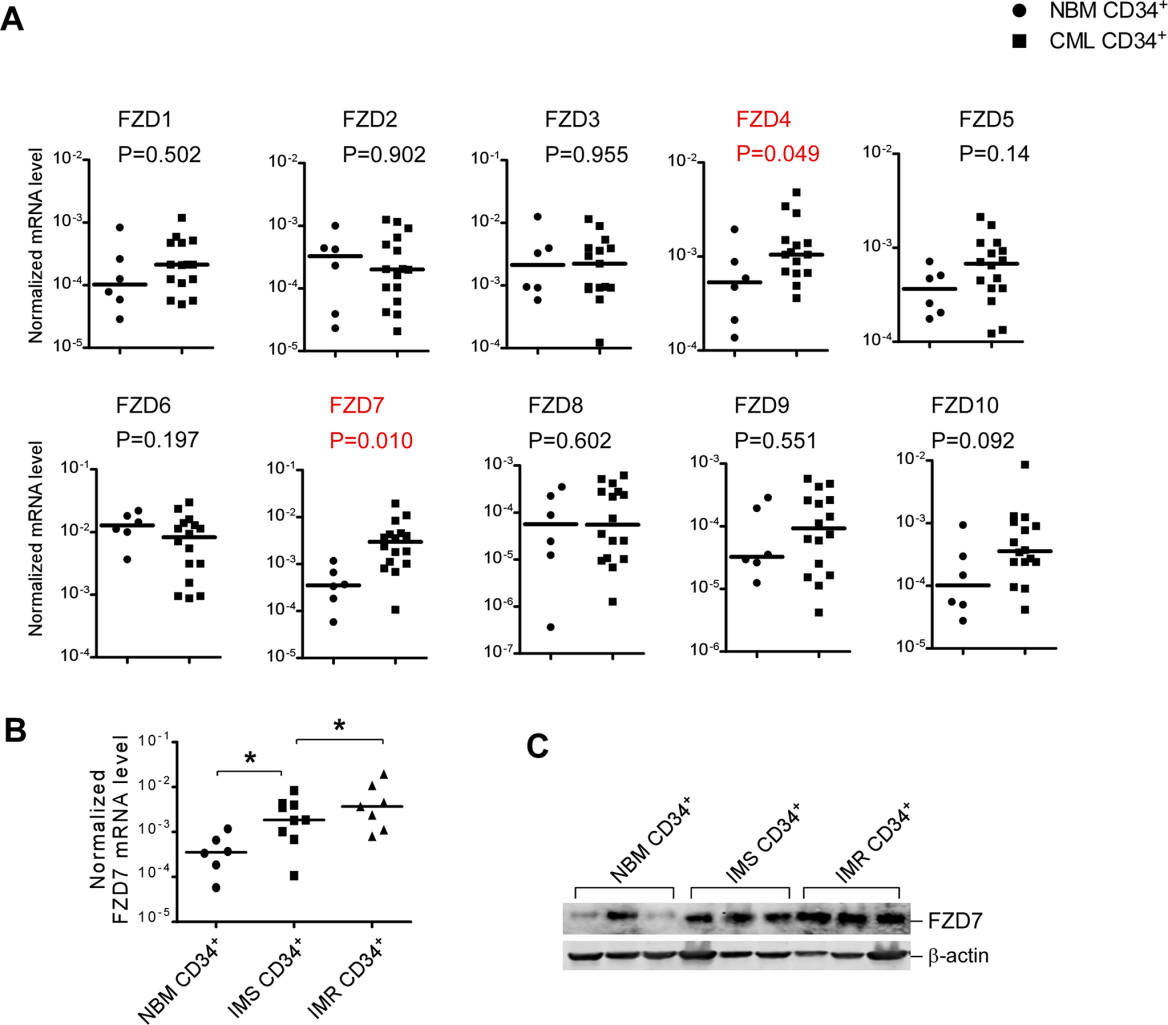
Several differentially expressed FZD genes identified in CML patients compared with normal stem/progenitor cells (**A**) Real-time RT-PCR analysis of expression levels of ten FZD receptors in untreated CML CD34^+^ cells (***n*** = 16) and NBM CD34^+^ cells (***n*** = 6). β-actin was used to normalize the mRNA level. Comparison of transcript levels of ten FZD genes in pre-treatment CML CD34^+^ cells NBM CD34^+^ cells. Solid points indicate individual values and horizontal lines represent the group median. (**B**) Comparison of transcript levels of FZD7 in CD34^+^ cells from NBM (*n* = 6), IM-sensitive patients (*n* = 9) and IM-resistant patients (*n* = 7). *P < 0.05. (**C**) Western blot analysis of FZD7 in CD34+ cells from NBM (*n* = 3), IM-sensitive patients (*n* = 3) and IM-resistant patients (*n* = 3).

As FZD7 was highly up-regulated when CML cells were co-cultured with BMSCs, we examined the potential role of FZD receptors in CML. First we investigated the mRNA levels of FZD family in primary CML CD34^+^ cells by real-time RT-PCR. In normal bone marrow (NBM) CD34^+^ cells, all FZD genes were detectable, but the expression level were variable between genes, with relatively highest expression level of *FZD6* and *FZD7*. More importantly, two FZD genes, *FZD4* and *FZD7* were differentially expressed in CML CD34^+^ cells compared to NBM CD34^+^ cells, while *FZD7* showed the highest elevation (Figure 2A).

To further confirm our results, relative *FZD7* mRNA levels of BMMCs from the 55 newly diagnosed adult CML patients and 20 healthy controls were also determined by real-time RT-PCR. In spite of the wide individual variance, mean levels of *FZD7* were significantly up-regulated in the CML patients, compared with the normal controls (*p* = 0.012) (Supplementary Figure S4).

### FZD7 is further up-regulated in IM-resistant CML CD34^+^ cells

To investigate the expression changes of FZD7 in response to TKIs therapy, we measured the mRNA and protein level of FZD7 in IM-sensitive (IMS) patients and IM-resistant (IMR) patients. As expected, *FZD7* mRNA levels showed higher expression level in CML CD34^+^ cells from IMR patients (*n* = 7) than IMS patients (*n* = 9) (Figure 2B). Western blot analysis also revealed that FZD7 protein levels were significantly elevated in IMR CML CD34^+^ cells, compared to their counterparts (Figure 2C). These findings raised the possibility that FZD7 could contribute to the drug resistance of IM in CML patients.

### Down-regulation of FZD7 suppressed the proliferation of CML cells

**Figure 3:**
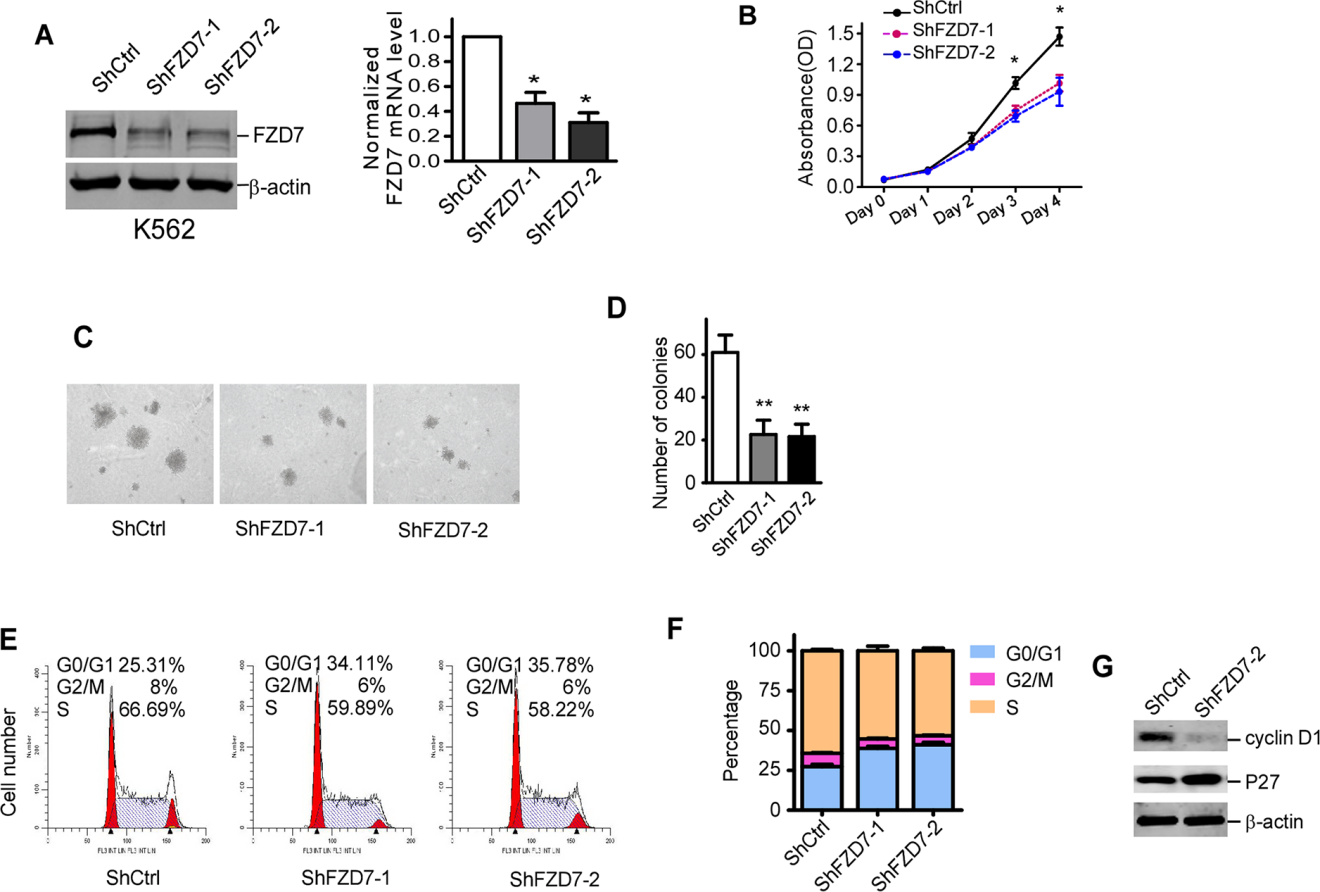
Down-regulation of FZD7 suppressed proliferation of K562 cells (**A**) After transduced with specific shRNA lentiviral particles for 3 days, K562 cells were analyzed by western blot and real-time RT-PCR for FZD7 protein and mRNA level, repectively. β-actin was used as internal control. For western blot, one representative figure from three experiments was shown. For real-time PCR, data were calculated from 3 experiments. Values represent means ± S.E. (*n* = 3). *P < 0.05. (**B**) K562 cells were transduced with specific FZD7 shRNA lentiviral particles for 3 days, and cell growth rate was analyzed by MTT assay. Data shown were average values of three independent experiments. *P < 0.05, **P < 0.01. (**C** and **D**) Soft agar colony formation of transduced K562 cells. Colonies > 0.1 mm in diameter were counted under a microscopic field at 50× magnifications. Two independent experiments were performed in triplicate. One representive picture was shown. Colums represented means ± S.E. *P < 0.05. (**E** and **F**) Transduced K562 cells were then stained with PI and analyzed for DNA content using FACS Calibur. The percentage of cells in G0/G1, S and G2/M of each group was shown in (F). Data plotted were means ± S.D. of three separate experiments. (**G**) Cell cycle regulators cyclin D1 and P27 were analyzed in transduced K562 cells. Results shown were representative of at least three independent experiments.

We supposed FZD7 was crucial in preventing the apoptosis of CML cells or promoting the proliferation of CML cells, thus loss of FZD7 should suppress the growth of CML cells. To test this possibility, we transduced CML cells with short hairpin RNAs that target *FZD7* (ShFZD7–1 and ShFZD7–2) or control (ShCtrl) for 3 days. Transfection efficiency was more tnan 90% evaluated by fluorescence microscope (Supplementary Figure S5). Real-time RT-PCR and western blot showed that both ShFZD7–1 and ShFZD7–2 could efficiently decrease FZD7 mRNA and protein level in K562 cells (Figure 3A). We observed that down-regulation of FZD7 suppressed cell growth of K562 cells, compared with negative control (Figure 3B). FZD7 knockdown also resulted less and smaller colony formation of K562 cells (61 ± 14.1 *versus* 22.6 ± 11.5 for ShFZD7–1, and 21.2 ± 10.1 for ShFZD7–2) (Figure 3C).

We next explored the underlying mechanisms of proliferative inhibition upon FZD7 down-regulation in CML cells by detecting the cell cycle with FACS. As shown in Figure 3E and 3F, down-regulation of FZD7 significantly increased the ratio of cells in G0/G1 phase, and simultaneously reduced the ratio of cells undergoing S phase. To further identified genes that are responsible for the cell cycle arrest caused by FZD7 knockdown, representative cell cycle regulators were studied. As shown in Figure 3G, p27 was significantly increased, while cyclin D1 was noticeably downregulated when K562 cells were treated with FZD7 shRNAs. These results provided evidences that FZD7 down-regulation suppressed proliferation of K562 cells by mediating G0/G1 arrest.

### Down-regulation of FZD7 sensitized CML cells to IM

**Figure 4:**
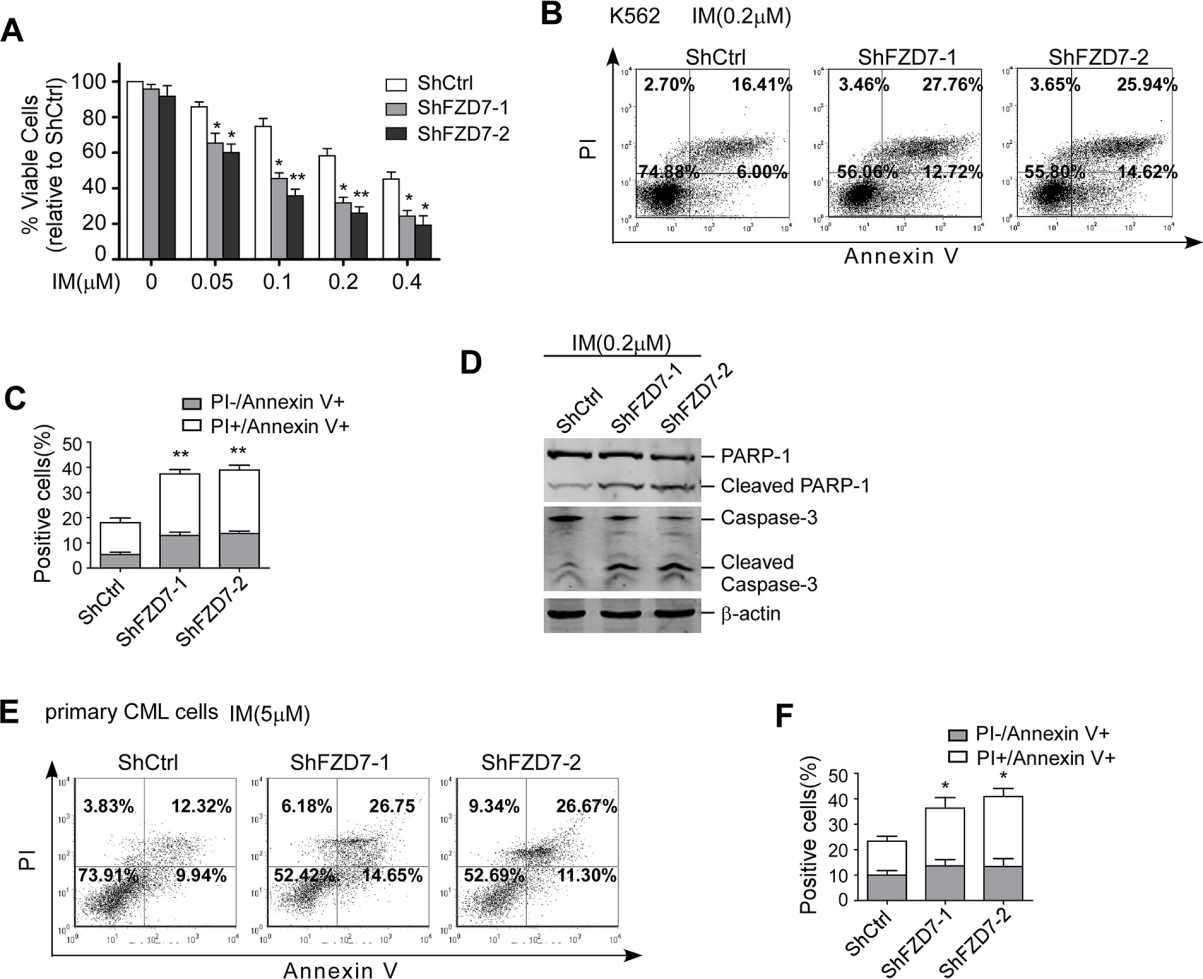
Down-regulation of FZD7 increased apoptosis induced by IM (**A**) After transduced with specific FZD7 shRNA lentiviral particles for 3 days, K562 cells were treated by imatinib with serial concentrations for additional 48 hrs. MTT assay was conducted to evaluate drug sensitivity. Results were average values of three independent experiments. *P < 0.05. (**B** and **C**) Transduced K562 cells were treated by IM (0.2 μM) for 48 hrs and subjected to Annexin V-APC/PI staining for flow cytometry. The percentage of cells was shown in each quadrant. Colums represented means ± S.E. **P < 0.01. (**D**) Transduced K562 were treated with IM for another 48 hrs. Caspase-3 and PARP-1 were estimated by Western blot. (**E** and **F**) Primary CMLcells were transduced with FZD7 shRNA lentiviral particles for 3 days, and then cultured with 5 μM IM for 48 hrs. Cells were subjected to annexin V-APC/PI staining for flow cytometry. The percentage of cells was shown in each quadrant. Colums represented means ± SE (*n* = 3). *P < 0.05.

As stated above, FZD7 levels were up-regulated in IM-resistant CD34^+^ CML cells. One possibility to explain this phenomenon is that FZD7 could be a negative regulator of the sensitivity of CML to IM. To test this possibility, we explored whether knockdown of endogenous FZD7 could reverse the IM-resistance induced by BMSCs in CML cells. As shown in Figure 4A, we found down-regulation of FZD7 enhanced the inhibitory effects of IM against K562 cells, and the IC50 (drug concentration leads to 50% decrease of cell viability) values of ShFZD7–1, ShFZD7–2 and ShCtrl were 0.103 ± 0.016, 0.072 ± 0.026 and 0.318 ± 0.056 μM, respectively (*P* < 0.01). Similarly, we also found ShFZD7 enhanced apoptosis induced by IM in K562 cells (Figure 4B and 4C). We further confirmed our results by detecting hallmarks of apoptosis, including the cleavage activation of caspase-3 and PARP-1. The cleaved caspase-3 and PARP-1 were significantly increased in K562 cells when FZD7 was knocked down (Figure 4D).

Primary cells from one CML patient was transduced with ShFZD7–1, ShFZD7–2 or ShCtrl and then treated with 5 μM IM for 48 hrs. The cell apoptosis was assessed by annexin V-APC/PI double-staining. The flow cytometry results showed that the percentage of apoptotic cells were 37.6 ± 8.09% and 40.9 ± 7.48% in ShFZD7–1 and ShFZD7–2 transduced cells, respectively, *versus* 22.3 ± 6.40% in ShCtrl transduced cells (*P* < 0.01, Figure 4E and 4F). Taken together, our results demonstrated that knockdown of FZD7 increased the sensitivity of CML cells to IM.

### Down-regulation of FZD7 abrogated IM resistance induced by BMSCs

**Figure 5:**
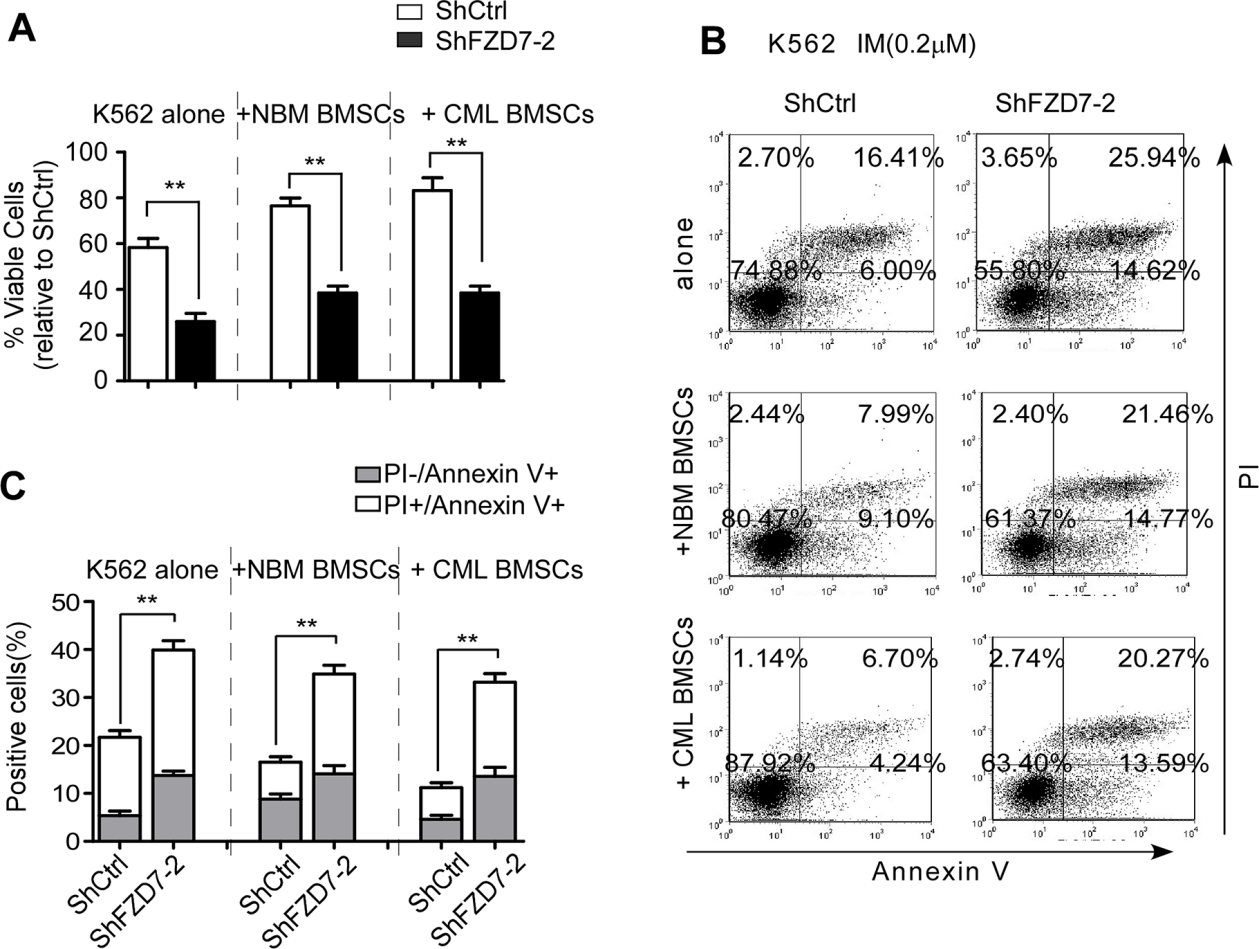
Down-regulation of FZD7 abrogates IM resistance induced by BMSCs K562 were transduced with ShCtrl or ShFZD7-2 lentiviral particles for 3 days, and then cultured solely or co-cultured with BMSCs (derived from CML patients or healthy donors) for additional 2 days. Adherent cells (co-cultured) were separated using a standardized wash procedure and treated by 0.2 μM IM for additional 48 hrs. (**A**) MTT assay was conducted to evaluate drug sensitivity. Results were average values of three independent experiments. *P < 0.05. (**B** and **C**) K562 cells were subjected to Annexin V-APC/PI staining for flow cytometry. The percentage of cells was shown in each quadrant. Colums represented means ± S.E. **P < 0.01.

To further study whether altered response of leukemic cells to IM by BMSCs was due to the activation of FZD7/Wnt/β-catenin signaling, FZD7 was down-regulated by shRNA lentiviral particles in K562 cells, and then cell survival of co-cultured K562 cells was examined. Control or FZD7 shRNA-transduced K562 cells were co-cultured with BMSCs and exposed to IM for 48 hrs. As shown in Figure 5A, K562 with FZD7 knocked down exhibited significantly higher susceptibility to IM compared with negative controls (*P* < 0.01). Next, we determined the effect of FZD7 on IM-induced apoptosis in co-cultured K562 cells by flow cytometry after annexin V/PI staining (Figure 5B). ShFZD7 or ShCtrl transduced-K562 cells were co-cultured with normal BMSCs, and exposed to IM for 48 hrs. The mean apoptotic rates of ShFZD7 or ShCtrl group were 35.1 ± 6.1% and 18.6 ± 4.1%, respectively. The mean apoptotic rates of K562 cells transduced with ShFZD7–2 and ShCtrl after treated with IM in the presence of CML BMSCs were 32.9 ± 6.1% and 13.1 ± 3.9%, respectively (Figure 5C). These results indicated that FZD7 down-regulation reversed IM resistance induced by BMSCs.

### Down-regulation of FZD7 inhibits Wnt/β-catenin signaling pathway and abrogates its activation induced by BMSCs

**Figure 6:**
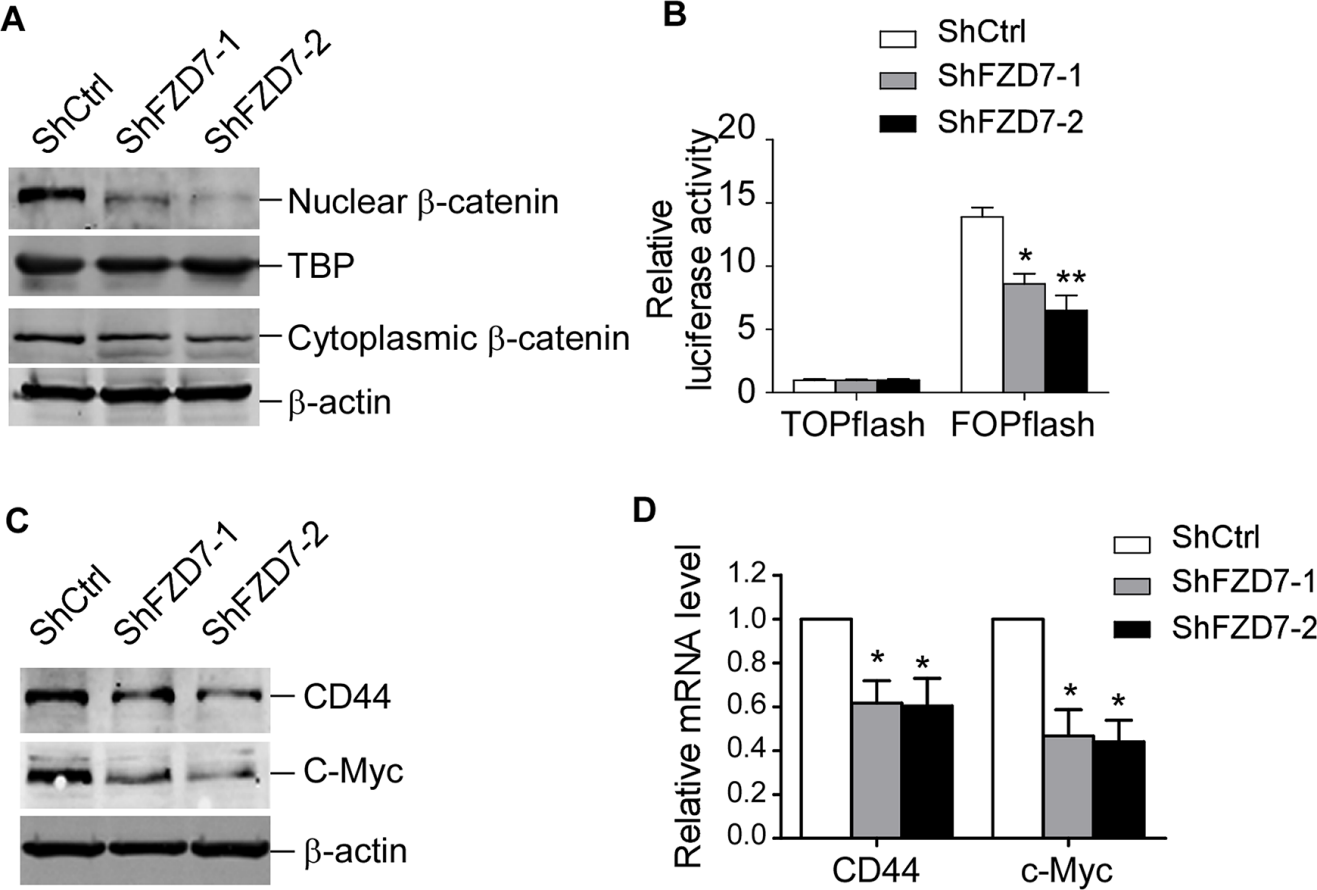
FZD7 knockdown inhibits Wnt/β-catenin signaling pathway K562 were transduced with ShCtrl, ShFZD7-1 or ShFZD7-2 lentiviral particles for 3 days. (**A**) Western blot analysis of β-catenin in nuclear and cytoplasm. TBP (TATA-box binding protein) and β-actin were used as internal control for nuclear and cytoplasmic protein, separately. Results shown were representative of three independent experiments. (**B**) TOPflash or FOPflash reporter plasmids were nucleofected into K562 cells after transduced with ShCtrl, ShFZD7-1 or ShFZD7-2 vectors. TCF reporter activity was measured by dual-luciferase assay. Relative luciferase expression levels were expressed as means ± SE (*n* = 3). *P < 0.05. **P < 0.01. (**C**) Western blot and (**D**) Real-time RT-PCR analysis of the Wnt pathway responsive genes c-Myc and CD44. Results shown were representative of three independent experiments. *P < 0.05.

FZD7 is known as the ligand receptor in the Wnt/β-catenin signaling pathway, which is involved in the chemoresistance of CML cells. We supposed that FZD7 effected on CML cells through Wnt/β-catenin signal pathway. Firstly we detected the activity of Wnt signaling following down-regulation of FZD7 in CML cells Our results showed that β-catenin in both nuclear and cytoplasm were attenuated by ShFZD7 comparing with ShCtrl (Figure 6A). TOPflash luciferase activity was also found to be markedly reduced by ShFZD7 compared to negative control (Figure 6B). We then assessed whether expression of the canonical Wnt pathway target genes (e.g. c-Myc and CD44) were impaired by FZD7 inhibition. Western blot analysis and RT-PCR revealed that the expression of CD44 and c-Myc in K562 cells were significantly decreased when FZD7 was knocked down (Figure 6C and 6D).

**Figure 7:**
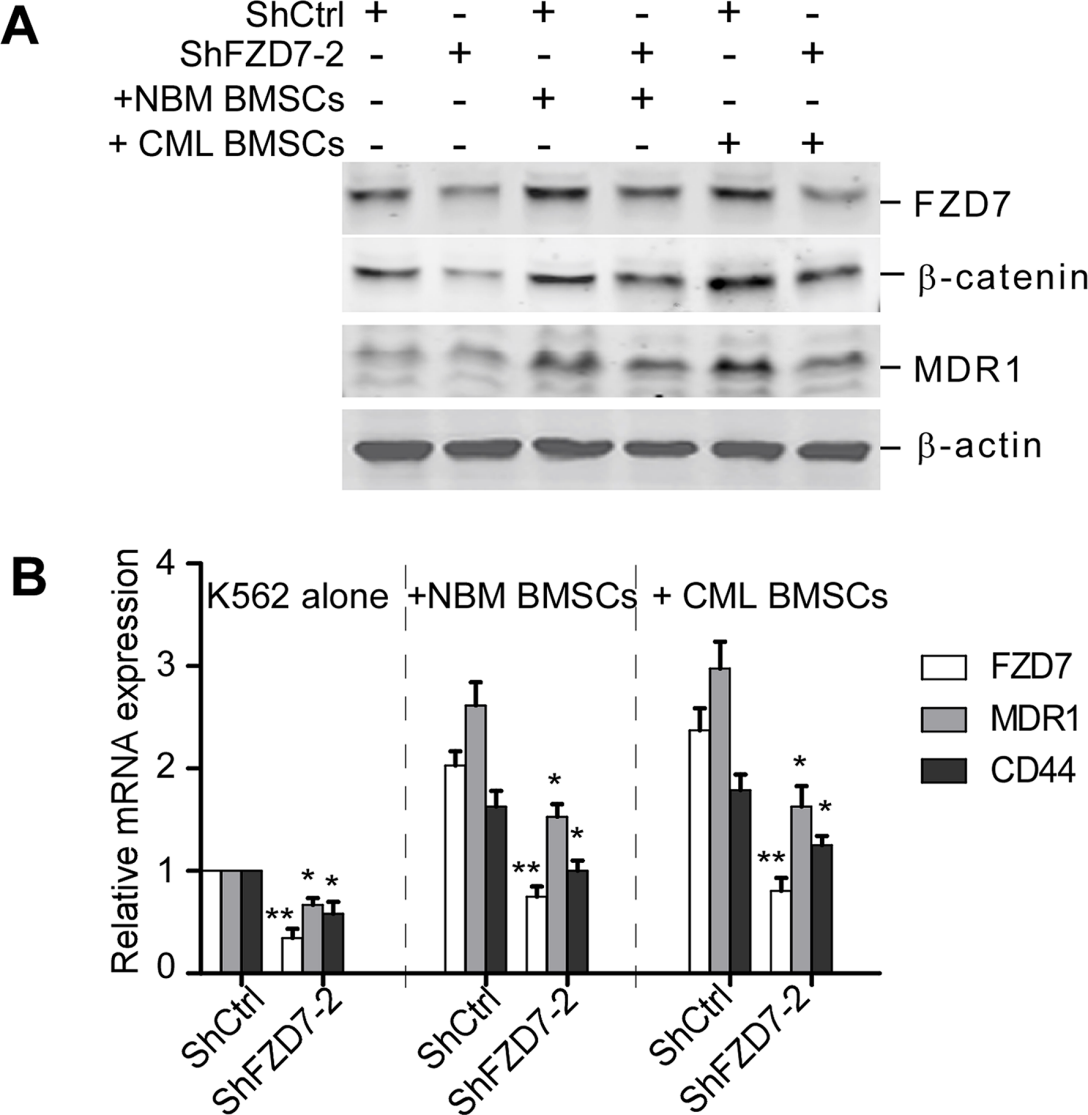
Down-regulation of FZD7 abrogates activation of Wnt/β-catenin signaling induced by BMSCs K562 were transduced with ShCtrl or ShFZD7-2 lentiviral particles for 3 days, and then cultured solely or co-cultured with BMSCs (derived from CML patients or healthy donors) for additional 2 days. K562 cells co-cultured were separated using a standardized wash procedure. (**A**) Western blot was used to detect the expression of FZD7, β-catenin and downstream Wnt downstream molecular-MDR1. (**B**) Real-time RT-PCR analysis was used to detect the expression of FZD7, as well as downstream Wnt downstream molecular-MDR1 and CD44. Values represent means ± S.E. (*n* = 3). *P < 0.05. **P < 0.01.

We next analyzed the possible mechanisms of restoration of sensitivity to chemotherapy of K562 cells by down-regulation of FZD7 following contact with BMSCs. Western blot results showed that when FZD7 was down-regulated in K562 cells following contact with BMSCs, the expression of FZD7, β-catenin, and MDR1 was still remarkably decreased (Figure 7A). we further examined the mRNA expression levels of *FZD7*, Wnt downstream gene *MDR1* and *CD44* in transduced K562 cells after co-culturing with CML or normal BMSCs. We found that *FZD7*, *MDR1* and *CD44* mRNA levels were markedly reduced by real-time qRT-PCR (Figure 7B). These results implied that FZD7 played an important role in BMSCs-mediated apoptotic resistance by activating Wnt/β-catenin signaling pathway.

## DISCUSSION

FZD7 has been demonstrated as a critical receptor of the Wnt signaling and involves in tumorigenesis and metastasis in many cancer types. The expression level of FZD7 was found up-regulated in several malignant tumors, such as colorectal cancer, hepatocellular carcinoma, esophageal cancer, lung cancer and gastric cancer, etc [14, 15]. Elevated levels of FZD7 protein in human breast cancer were associated with unfavorable prognosis and progressive stages of disease [16, 17]. In our study, we found that the expression of FZD7 was up-regulated in CML patients compared with normal controls. What's more, the expression level of FZD7 in IMR CML cells was significant higher that IMS cells, suggesting that FZD7 may take part in the drug resistance of CML. More interestingly, FZD7 was up-regulated by BMSCs, and BMSCs from CML patients showed increased efficiency to up-regulate the expression of FZD7 along with increment of β-catenin and Wnt target molecules such as MDR1, c-Myc, and CD44 in CML cells. As we have known that the interaction between BMSCs and leukemia cells may trigger molecular changes of some important signal pathways that lead to leukemia, such as Notch signaling [18]. This is the first experimental evidences that signals from the stromal microenvironment induce FZD7 expression in CML cells. Our data indicated that external signals from BMSCs may have an important role in the preserve of CML cells.

FZD7 is reported to promote the growth of several tumors. In hepatocellular carcinoma cells, the functional interaction between Wnt3 and FZD7 enhances proliferative rate [19]. In triple negative breast cancer, FZD7 shRNA reduces cell proliferation and colony formation [20]. In *Xenopus laevis*, different thresholds of Wnt-FZD7 activity coordinate with progenitor cell fate and proliferation rate of endoderm progenitor cells [21]. As FZD7 was up-regulated in CML cells, we down-regulated its expression by shRNA to evaluate the biological effect. We found that down-regulation of FZD7 impaired cell proliferation in CML cell line by increasing G0/G1 phase and decreasing S phase cells, and restored the sensitivity of CML cells to IM. It was the first time that the effect of FZD7 had been explored in CML. We further found knockdown of FZD7 in CML cells decreased protein level of β-catenin, especially in the nuclear localization, inhibiting *TCF* luciferase activity, suggesting the following event responsible for the effect.

Targeted therapy of CML using TKI have greatly improved the survival of patients. However, it remains incurable. Chomel *et al.* demonstrated the long-term persistence of a considerable amount of BCR-ABL-expressing stem cells even in the absence of relapse [22]. LSCs are known to be genetically unstable and less responsive to TKI treatments, and are of critical importance in mediating TKI resistance [23]. The bone marrow microenviroment contributes to the persistence of LSCs. The bone marrow niche may provide survival factors to CML stem cells in the context of LSC/niche cross-talk. For example, Zhang *et al.* found that N-cadherin–mediated adhesion played an important role in protection of CML stem/progenitor cells from TKI treatment by BM MSCs [12]. Recently the contribution of leukemia-induced alterations in the BM microenvironment also attracts lots of attention. CML BMSCs were demonstrated to reduce the expression of normal BM, influenced the normal stem cell niche, causing alteration in the microenvironmental factors [24, 25]. Leukemia BM decreased CXCL12 expression and up-regulated G-CSF, IL-1α, MIP-1β, and MIP-2, compared to normal BMSCs [26]. In addition, mice with BCR-ABL-transformed leukemia exhibit analogous alterations of BMSCs and osteoblastic cells along with their supporting effects on leukemogenesis [27, 28]. These studies give rise to the concept of “leukemia-induced microenvironment” [29]. Here, we also found an interaction between the bone marrow micrienviroment and leukemia cells partially mediated by FZD7. BMSCs from CML patients was shown to be more efficient to up-regulate FZD7/β-catenin siganling in CML cells than normal BMSCs, thus to accelerate proliferation, enhance IM resistance. This indicated that remodeled BMSCs by leukemia provided more survival signals to fuel the growth of leukemia cells and contribute to the IM resistance. Some scientists are trying to overcome IM resistance by targeting oncogenic kinases by different mechanisms such as combined TKI with geldanamycin(GA) or JAK2 inhibitor [30, 31]. Our experiment may provide another approach by combination of TKI with FZD7 inhibitor.

The Wnt/β-catenin signaling pathway plays important role in pathogenesis and progression of CML. Increased activity of Wnt/β-catenin pathway was correlated with poor response to imatinib and LSCs transformation in blast crisis CML [32, 33]. In addition, β-catenin deletion in mice caused a profound reduction in the risk of CML induced by BCR-ABL [34]. Hu *et al.* found that β-catenin was essential for survival and self-renewal of LSCs [35]. Genetic and pharmacologic inhibition of b-catenin contributed to the apoptosis of imatinib-resistant leukemia stem cells in CML [36]. Potential mechanisms underlying increased b-catenin in CML cells include BCR-ABLmediated b-catenin phosphorylation [37] and GSK3β inactivation [38], leading to protein stabilization of β-catenin. Here we reported a new mechanism in regard to the activation of Wnt/β-catenin signaling pathway in CML. We found FZD7 was overexpressed in CML cells in a co-culture system with BMSCs cells, resulting in up-regulated b-catenin, activated Wnt signaling and increased resistance to IM, which could be eliminated by antagonism of FZD7 expression. Our study suggested that FZD7 played an important role in the cross-talk between BMSCs and CML cells, indicating FZD7 could be a potential therapeutic target for CML.

## MATERIALS AND METHODS

### Material and cell lines

K562 cells were cultured in RPMI 1640 medium supplemented with 10% fetal bovine serum (Gibco, Grand Island, NY) and 1% penicillin-streptomycin in an incubator maintained at 37°C in an atmosphere containing 5% CO_2_. Primary antibodies for FZD7 were purchased from Abcam (Austin, TX), those for β-catenin, MDR1, caspase 3, and cyclin D1 were purchased from CST (Beverly, MA), that for b-actin was from Sigma-Aldrich (St Louis, MO), those for PARP-1 and P27 were from Santa Cruz (Santa Cruz, CA). All secondary antibodies were obtained from Li-Cor Biosciences (Lincoln, USA).

### Clinical samples

Bone marrow mononuclear cells (BMMCs) of 55 initially diagnosed CML patients and 20 healthy donors were obtained after informed consent at Qilu Hospital of Shandong University from May 2011 to December 2012. The detailed clinical information of the 55 patients is are available in Supplementary Table S1. Bone marrow samples from another 16 newly diagnosed CML patients prior to TKIs therapy and 6 healthy donors were taken after informed consent. Subsequent imatinib sensitive (IMS, *n* = 9) patients achieved complete hematologic remission within 3 months, major cytogenetic remission within 12 months, and complete cytogenetic remission within 18 months, based on the European Leukemia Net treatment guidelines [13]. Conversely, imatinib resistant (IMR, *n* =7) patients did not achieve these response criteria or had evidence of loss of response later. Mononuclear cells were isolated by Ficoll-Hypaque (Sigma-Aldrich, St Louis, MO) density gradient centrifugation and enriched using a human CD34 MicroBead Kit (Miltenyi Biotec, Germany). All the study protocols involved with patients and healthy donors were approved by the Medical Ethics Committee of Qilu Hospital of Shandong University, Jinan, China, and written informed consents were obtained from all patients and healthy donors.

### BMSCs and co-culture

Bone marrow samples used for BMSCs cultures were aspirates of healthy donors and CML patients with written informed consent. Mononuclear cells obtained after Ficoll-Hypaque density centrifugation were suspended in MesenPro RS Medium (GIBCO BRL) at 37°C and 5% CO_2_. BM non-adherent cells were removed after 1 days and the culture medium was replaced every 3–4 days until a confluent monolayer had developed (usually after 1–2 weeks). Passage 3 or 4 BMSCs were used for the coculture experiments. Adherent BMSCs were washed with FBS free medium, and K562 or primary CML cells were seeded onto BMSC monolayers at 4 × 10^6^ cells per mL in RPMI 1640. Co-cultured K562 cells were separated from BMSCs using a standardized wash procedure. Detail methods was shown in the Supplementary Data.

### Plasmids and lentiviral particles production

The target shRNA sequences for *FZD7* were chemically synthesized and cloned into pLVTHM lentiviral vector. Hek293T cells were co-transfected with a mixture of pLVTHM-shFZD7–1, pLVTHM-shFZD7–2 or pLVTHM, and psPAX2, pMD2.G to package lentiviral particles. Detailed methods were shown in the Supplementary Data.

### Colony formation in soft agarose gel, MTT assay, cell cycle analysis and apoptosis assay

Detail methods were shown in the Supplementary Data.

### Quantitative real-time RT-PCR

TRIzol reagent (Invitrogen, Carlsbad, CA) was applied to extract total RNA from cells or patient samples. Reverse transcription was performed using M-MLV reverse transcriptase cDNA Synthesis Kit (Takara, Japan). Real-time RT-PCR was carried out on ABI 7900HT Fast Real-Time PCR System (Foster City, CA) with SYBR-Green PCR Master Mix (Toyobo, Japan). Melting curves analysis was applied to guarantee the specificity of amplification. A comparative CT method (2^−ΔΔCT^) was used to analyze the gene expression level. β-actin or *GAPDH* was used as the internal control. The primers for real-time RT-PCR are available in Table 1.

**Table 1:** The primers for real-time RT-PCR

Name	Forward primer	Reverse primer
FZD1	5′-ATCTTCTTGTCCGGCTGTTACA-3′	5′-GTCCTCGGCGAACTTGTCATT-3′
FZD2	5′-GTGCCATCCTATCTCAGCTACA-3′	5′-CTGCATGTCTACCAAGTACGTG-3′
FZD3	5′-GTTCATGGGGCATATAGGTGG-3′	5′-GCTGCTGTCTGTTGGTCATAA-3′
FZD4	5′-GTCTTTCAGTCAAGAGACGCTG-3′	5′-GTTGTGGTCGTTCTGTGGTG-3′
FZD5	5′-CATGCCCAACCAGTTCAACC-3′	5′-CGGCGAGCATTGGATCTCC-3′
FZD6	5′-GCGATAGCACAGCCTGCAATA-3′	5′-AATGGTAAGAATCACCCACCAC-3′
FZD7	5′-GTGCCAACGGCCTGATGTA-3′	5′-AGGTGAGAACGGTAAAGAGCG-3′
FZD8	5′-ATCGGCTACAACTACACCTACA-3′	5′-GTACATGCTGCACAGGAAGAA-3′
FZD9	5′-TGCGAGAACCCCGAGAAGT-3′	5′-GGGACCAGAACACCTCGAC-3′
FZD10	5′-GCTCATGGTGCGTATCGGG-3′	5′-GAGGCGTTCGTAAAAGTAGCA-3′
MDR1	5′-TTGCTGCTTACATTCAGGTTTCA-3′	5′-AGCCTATCTCCTGTCGCATTA-3′
c-Myc	5′-TCAAGAGGTGCCACGTCTCC-3′	5′-TCTTGGCAGCAGGATAGTCCTT-3′
CD44	5′-CTGCCGCTTTGCAGGTGTA-3′	5′-CATTGTGGGCAAGGTGCTATT-3′
Trib2	5′-CTTTTGCCTGTCTGCTCATAGT-3′	5′-ATAGCTTCGCTCAAAGAACACA-3′
Survivin	5′-GCTTTCAGGTGCTGGTAG-3′	5′-GATGTGGATCTCGGCTTC-3′
β-actin	5′-CACTGTGTTGGCGTACAGGT-3′	5′-TCATCACCATTGGCAATGAG-3′
GAPDH	5′-CTGGGCTACACTGAGCACC-3′	5′-AAGTGGTCGTTGAGGGCAATG-3′

### Western blot

The extraction and isolation of nuclear and cytosolic protein were by Nuclear and Cytoplasmic Protein Extraction Kit (Beyotime, China) according to the manufacturer's instructions. For western blot analysis, cells were harvested by centrifugation and washed twice with phosphate-buffered saline (PBS), and solubilized in RIPA lysis buffer (Beyotime, China) containing protease inhibitor cocktail (Roche, Indianapolis, IN). 30μg protein sample were separated on 10% SDS–polyacrylamide gel and transferred onto nitrocellulose membranes. Membranes were blocked in 3% non-fat milk for 1 hour and incubated in primary antibodiesovernight. After secondary antibody incubation, signals were detected and analyzed by the Li-Cor Odyssey imaging system (Lincoln, NE).

### Statistical analysis

Student's *t* test was used for statistical comparison between groups. or patient samples, *FZD7* mRNA level was presented quantitatively as median. The difference in the newly diagnosed patients and normal controls was performed using a one-way ANOVA test. The statistical analysis was performed using SPSS 17.0 software.

## SUPPLEMENTARY MATERIALS FIGURES AND TABLE


