# Supplementary material for: FZD7 regulates BMSCs-mediated protection of CML cells
Source: Oncotarget. 2015 Dec 23;7(5):6175–87. doi: 10.18632/oncotarget.6742 (PMC4868748; doi:10.18632/oncotarget.6742)
Supplement: Supplementary file 1 [file oncotarget-07-6175-s001.pdf]

# FZD7 regulates BMSCs-mediated protection of CML cells

## Supplementary Materials

### Co-culture system

For co-culture experiments, BMSCs were seeded at 3 × $10^5$ cells/mL and incubated overnight. Adherent BMSCs were washed with FBS free medium, and K562 or primary CML cells were seeded onto BMSC monolayers at 4 × $10^6$ cells per mL in RPMI 1640, 1% penicillin/streptomycin and 10% FBS. Co-cultured K562 cells were separated from BMSCs using a standardized wash procedure. First, the culturing plates were shaken gently and the supernatant was collected into 15-mL tubes. Add a certain volume of medium into the wells and repeat the procedures above for another two times. Check the culturing plates under a microsope to make sure that the adhering BMSCs were not disturbed (Figure S1B), comparing to BMSCs cultured alone (Figure S1C) and the majority of K562 cells were collected for following experiments. Because the K562 cells were infected with PLVTHM letiviral particles which contains a GFP tag before co-culturing with BMSCs, we perormed flow cytometry to assess BMSC contamination in harvested K562 cells. Figure 2A shows that un-transduced K562 cells exhited no GFP staining (Figure S2A) and 96.7% PLVTHM transduced cells was GFP-positive (Figure S2B), whereas the GFP-positive rate of collected K562 cells drop to 95.2% (Figure S2C). Based on these data, we speculate that the contaminated BMSCs account for less than 1.5% in the K562 cells collected from the co- culture system (Figure S2).

### Plasmids and lentiviral particles production

The target shRNA sequences for *FZD7* were chosen by the Ambion's siRNA Target Finder, and were subjected to Basic Local Alignment Search Tool (BLAST) search to ensure that there was no sequence homology to other genes. ShRNA oligonucleotides with MluI and ClaI restriction sites in the 5′ and 3′ ends were chemically synthesized: FZD7 shRNA-1: 5′- CGC GTC CCC TCA CCT ACC TGG TGG ACA TGC TTC AAG AGA GCA TGT CCA CCA GGT AGG TGA TTT TTG GAA AT -3′; FZD7 shRNA-2: 5′-CGC GTC CCC GGC CTG ATG TAC TTT AAG GAT TTC AAG AGA ATC CTT AAA GTA CAT CAG GCC TTT TTG GAA AT -3′. FZD7-specific short hairpin oligonucleotides were annealed and cloned into pLVTHM lentiviral vector. To package FZD7 shRNA lentiviral particles, Hek293T cells were co-transfected with a mixture of 10 μg pLVTHM-shFZD7-1, pLVTHM-shFZD7-2 or pLVTHM, and 6.67 μg psPAX2, 3.3 μg pMD2.G utilizing Lipofectamine 2000 (Invitrogen, Carlsbad, CA) according to the manufacturer's protocol. 48, 72 and 96 hrs after transfection, supernatants were collected and then concentrated with PEG8000.

### MTT assay

Briefly, cells were cultured in 96-well plates and incubated with 10 μL MTT (5 mg/mL) at 37°C for 4 hrs, and then 100 μL 10% SDS (PH 4.0) was added to each well and incubated over night. The absorbance was measured at 570 nm. Each sample was performed in triplicates.

### Cell cycle analysis

Cell cycle analysis was performed using the cell cycle kit (BestBio, Shanghai, China), according to the manufacturer's instructions. Samples were analyzed by FACS (Beckman, CA).

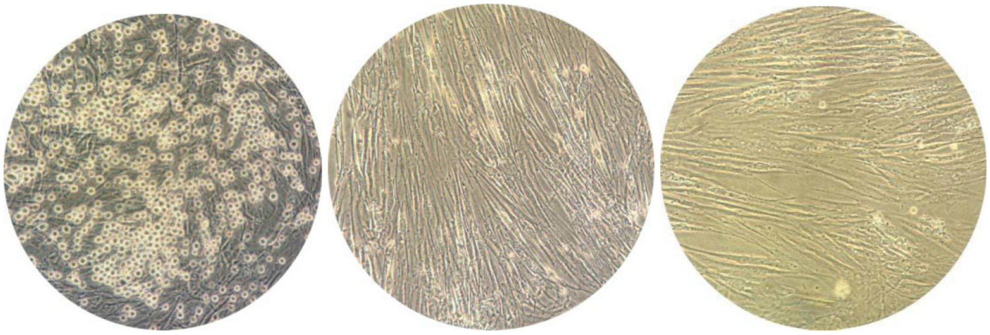

**Supplementary Figure S1:** (**A**) BMSCs were co-cultured with K562 cells. (**B**) After the collection of K562 cells, the adhering BMSCs were not disturbed. (**C**) BMSCs were cultured alone.

## Apoptosis assay

The apoptosis assay was performed using Annexin V-APC/PI apoptosis detection kit (Ebioscience, San Diego, CA), according to the manufacturer's instructions. Fluorescence of at least 10,000 cells was determined on a FACS Calibur flow cytometer to determine the percentage of apoptotic cells.

## Isolation of CD34$^+$

CD34$^+$ hematopoietic cells were isolated from bone marrow samples using the CD34 MicroBead Kit (Miltenyi Biotec) according to the manufacturer's instructions as following:

1. Determine cell number.
2. Centrifuge cell suspension at 300 × g for 10 minutes. Aspiratesupernatant completely.
3. Resuspend cell pellet in 300 µL of buffer for up to $10^8$ totalcells.
4. Add 100 µL of FcR Blocking Reagent for up to $10^8$ total cells.
5. Add 100 µL of CD34 MicroBeads for up to 108 total cells.
6. Mix well and incubate for 30 minutes in the refrigerator(2−8°C).
7. Wash cells by adding 5–10 mL of buffer for up to 108 cellsand centrifuge at 300 × g for 10 minutes. Aspirate supernatantcompletely.
8. Resuspend up to $10^8$ cells in 500 µL of buffer.
9. Proceed to magnetic separation:

(1) Place column in the magnetic field of a suitable MACSSeparator. For details refer to the respective MACS Columndata sheet.

(2) Prepare column by rinsing with 500 µL buffer:

(3) Apply cell suspension onto the column. Collect flow-throughcontaining unlabeled cells.

(4) Wash column with 3 × 500 µL buffer. Collectunlabeled cells that pass through and combine with the flowthroughfrom step 3.

(5) Remove column from the separator and place it on a suitablecollection tube.

(6) Pipette 1 mL buffer onto the column.Immediately flush out the magnetically labeled cellsby firmly pushing the plunger into the column.

(7) To increase the purity of CD34$^+$ cells, the elutedfraction can be enriched over a second MS Column. Repeat the magnetic separation procedure as described insteps 1 to 6 by using a new column.

## Luciferase assays

The TOPFlash construct contains a combinationof TCF binding sites placed upstream of luciferasecDNA. The negative control FOPFlash construct containsmutated TCF binding sites. Renilla luciferase pRL-TK was cotransfectedas an internal control for transfection efficiency.Transfections were performed using a Nucleofector (Amaxa) according to the manufacturer's instructionswith minor modifications. Briefly, 1 × 106 K562 cellstransduced with short hairpin RNAs that target FZD7 or control were transfected with 2.5 µg of either TOPFLASH or FOPFLASH along with 0.25 µg of Renilla cytomegalovirus (CMV) construct. After 24 hours, cell lysates were prepared and reporter activity was measured using the Dual-LuciferaseReporter Assay System (Promega).V

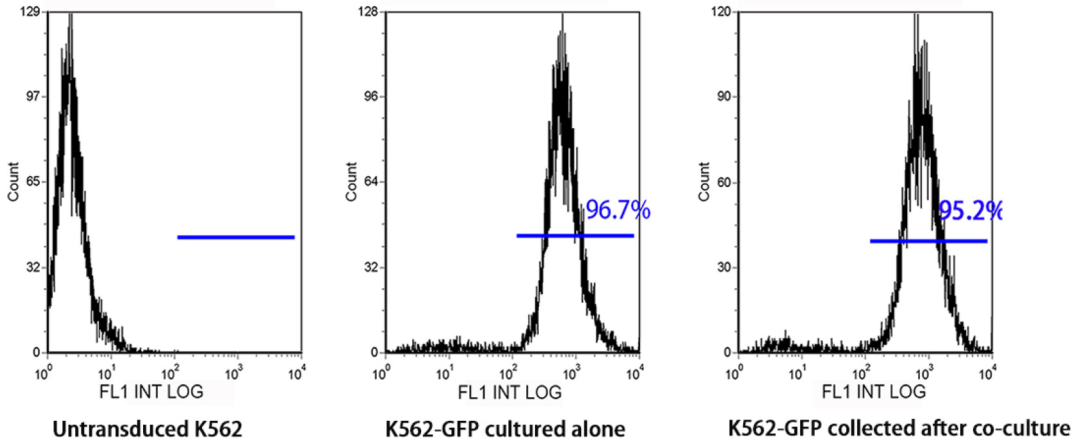


**Supplementary Figure S2: Less than 1.5% of BMSCs were contaminated in collected K562 cells.**

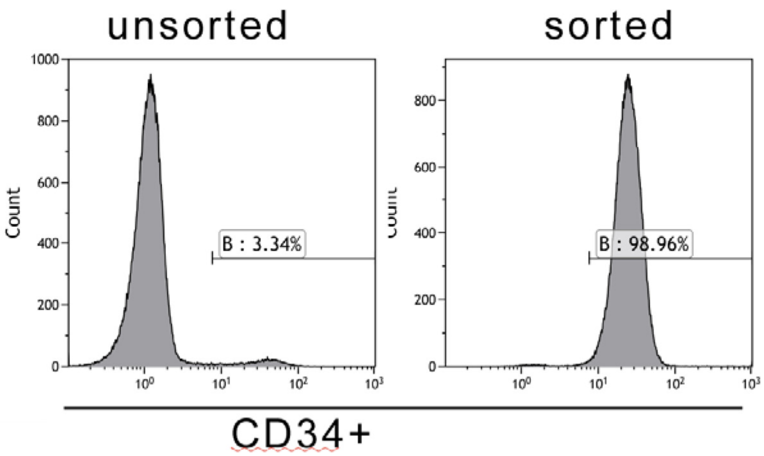


**Supplementary Figure S3: Flow cytometry shows that the percentage of CD34⁺ positive mononuclear cells in bone marrow significantly increases from 3.34% to 98.96% after magnetic sorting.**

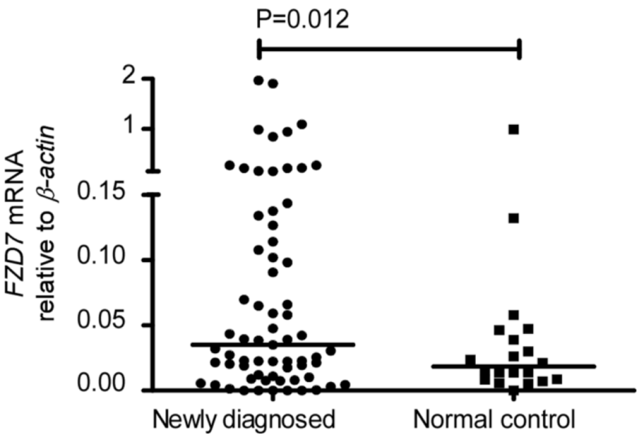


**Supplementary Figure S4: Real-time RT-PCR analysis of FZD7 expression in 55 untreated CML patients and 20 healthy controls.** Solid points indicate individual values and horizontal lines represent the group median. Data was analyzed using the Mann-Whitney *U* test.

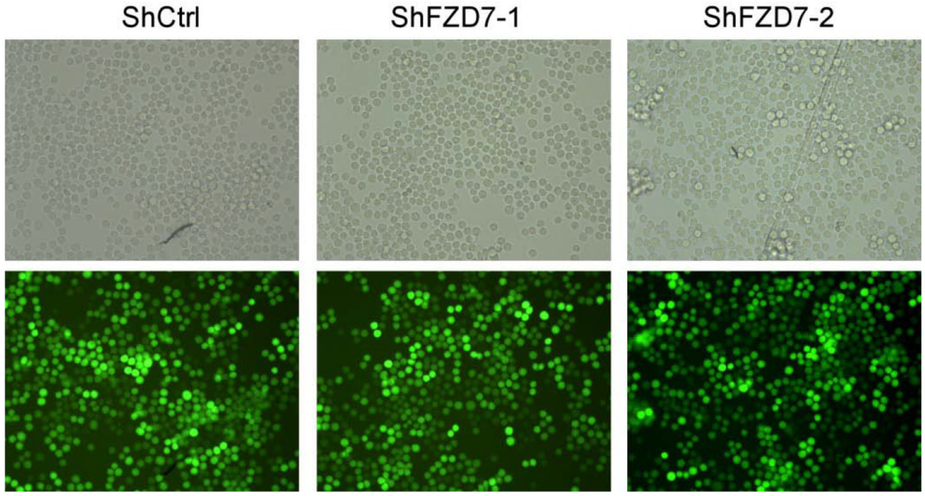


**Supplementary Figure S5: After K562 cells were transduced with specific shRNA lentiviral particles for 3 days, fluorescent microscope was used to the efficiency of transduction.**

**Supplementary Table S1: Characteristics of the 55 CML patients**

| No. of patients | 55 |
|---|---|
| Sex<br>male<br>female | <br>31<br>24 |
| Age: median (range)<br>Splenomegaly (%) | 42 (17–64)<br>72.9% |
| WBC (x$10^9$/l): median (range) | 147 (20.4–450) |
| Neutrophils (%): median (range) | 123.2 (13.72–362.58) |
| Hemoglobin (g/l): median (range) | 99 (48–161) |
| PLT (x$10^9$/l): median (range)<br>BM blast (%) median (range) | 374 (76–1821)<br>3 (1–12) |

Abbreviations: WBC, white blood cells; PLT, platelets; BM, bone marrow.
